# Microhabitat Selection Drives Tick Avoidance in Giant Pandas During Semi-Wild Adaptation Training: Implications for Conservation Management

**DOI:** 10.3390/ani16193050

**Published:** 2026-09-28

**Authors:** Rui Ma, Xiang Yu, Chong Huang, Fei Xue, Yanshan Zhou, Jiabin Liu, Rong Hou, Wenlei Bi, Jiang Gu, Long Zhang, Feifei Feng, Guanwei Lan, Zusheng Li, Wei Wu, Hong Yang, Dunwu Qi

**Affiliations:** 1 The Conservation of Endangered Wildlife Key Laboratory of Sichuan Province, Chengdu Research Base of Giant Panda Breeding, Chengdu 510081, China; marui_pandabase@outlook.com (R.M.);; 2 Daxiangling Nature Reserve, Yaan 625000, China

**Keywords:** *Ailuropoda melanoleuca*, animal welfare, enclosure design, microhabitat selection, parasite avoidance, pre-release management

## Abstract

Managing endangered wildlife in pre-release or semi-wild training programs requires an understanding of how animals balance strict energetic constraints against environmental health risks. We investigated the behavioral and spatial coping mechanisms of giant pandas (*Ailuropoda melanoleuca*) during semi-wild adaptation training exposed to intense seasonal tick (*Ixodes ovatus*) parasitism. Integrating high-resolution 3D-GPS telemetry, fine-scale microhabitat architecture mapping, and longitudinal daily tick counts, we evaluated the relative efficacy of behavioral hypoactivity versus microhabitat selection using Generalized Estimating Equations (GEE). During the high-risk summer season, individuals exhibited pronounced baseline behavioral suppression. However, GEE models revealed that fine-scale microhabitat selection—rather than localized movement reduction—was the primary determinant of parasite avoidance. Actively shifting to higher-elevation microhabitats with minimal leaf litter and dense bamboo coverage significantly minimized daily tick burdens. Conversely, remaining sedentary within low-elevation, thick-litter zones yielded the highest predicted parasite loads. These findings demonstrate that physical stillness alone is insufficient to mitigate ectoparasite exposure without access to structural habitat refugia. For conservation and reintroduction programs, providing structurally complex, topographically heterogeneous enclosures is essential. Ensuring access to natural altitudinal gradients empowers managed herbivores to autonomously regulate parasitological risks, thereby enhancing pre-release animal welfare and health.

## Simple Summary

Preparing endangered animals like giant pandas for release into the wild is challenging, particularly when they face severe tick infestations. In fenced training enclosures, animals cannot migrate long distances to escape these parasites. This study investigated how giant pandas adapt their behavior and habitat choices to reduce tick exposure during the high-risk summer months. By tracking pandas and monitoring their daily tick counts, we discovered that during peak tick season, the animals intentionally shifted their habitat use. They moved away from lower-elevation woodlands with thick leaf litter, preferring higher-elevation, cooler areas dominated by dense bamboo. They also reduced their daily travel distances. These findings demonstrate that giant pandas use precise habitat selection to actively avoid parasites. For wildlife conservation, this highlights the critical need to design pre-release enclosures that include diverse natural landscapes, such as high-elevation bamboo patches. Providing these varied environments allows endangered animals to naturally manage parasite pressures, ultimately improving their health, welfare, and success upon final release into the wild.

## 1. Introduction

Animals continuously balance the acquisition of essential nutritional resources against the necessity of avoiding environmental risks to maintain health and survival [1,2]. While behavioral research has predominantly focused on predator-driven spatial avoidance, parasite-driven spatial avoidance has emerged as a crucial determinant of animal distribution and behavioral plasticity [3,4,5]. Parasites impose severe physiological costs on hosts, driving the evolution of highly sensitive detection and avoidance mechanisms [6]. For instance, domestic sheep (*Ovis aries*) actively avoid grazing near feces-contaminated pastures to minimize gastrointestinal nematode infection [7], while mandrills (*Mandrillus sphinx*) rely on olfactory signals to maintain spatial distance from parasitized group members [8,9]. While these well-documented phenomena highlight the behavioral avoidance of infected conspecifics, animals must also navigate and actively avoid environmentally mediated risks, such as specific microhabitats harboring high densities of free-living parasitic stages or vectors [7,10].

To mitigate environmental parasite exposure, wild animals have evolved a diverse repertoire of behavioral and spatial strategies [11]. For example, North American bison (*Bison bison*) adjust their diurnal grazing schedules and utilize wallowing behaviors to minimize exposure to questing ticks in low-lying vegetation [12,13]. In alpine and Arctic ecosystems, reindeer (*Rangifer tarandus*) abandon high-biomass foraging valleys during summer and migrate to high-elevation snow patches or wind-exposed ridges to escape severe harassment by parasitic oestrid flies, explicitly trading optimal nutrition for parasitological relief [14,15]. Despite these well-documented phenomena, a critical knowledge gap remains in applied animal behavior and conservation: most empirical studies evaluate macro-spatial relocation and behavioral state modification as isolated responses [16]. Few investigations have quantitatively deconstructed the synergistic interactions between fine-scale microhabitat selection and concurrent behavioral suppression, particularly when environmental parasite pressure reaches seasonal saturation [17,18].

The giant panda (*Ailuropoda melanoleuca*) undergoing semi-wild adaptation training provides an exceptional model to investigate these behavioral and physiological trade-offs in a managed conservation context [19]. Having evolved a highly specialized ecological niche, giant pandas exhibit extremely efficient microhabitat utilization to sustain their basal requirements [20,21]. Despite this adaptive efficiency, field investigations reveal that both wild and managed giant pandas experience severe tick infestations during the summer months [22,23]. Depending on the specific geographic region, these hosts are exposed to various tick species, predominantly belonging to the genera *Ixodes*, *Haemaphysalis*, and *Dermacentor*, which function as primary vectors for numerous zoonotic pathogens [24,25,26,27]. As obligate herbivores reliant on a bamboo diet with extremely low energy conversion efficiency, giant pandas maintain an exceptionally low daily energy expenditure [19,28]. Consequently, undertaking macro-geographical migrations to escape seasonal parasite exposure is energetically prohibitive, a constraint further amplified within fenced pre-release enclosures [29]. To maintain health and welfare under such severe metabolic constraints, giant pandas must achieve a precise balance between energy conservation and parasite avoidance within their restricted spatial environments [30,31].

Our study investigates the fine-scale spatiotemporal responses of giant pandas during semi-wild adaptation training to peak seasonal tick exposure. By integrating continuous high-resolution 3D-GPS telemetry, comprehensive microhabitat surveys, and longitudinal parasite load data, we aim to quantify the relative contributions and synergistic interactions of spatial and behavioral plasticity. We hypothesize that giant pandas employ a dual-layered defense strategy—comprising spatial shifts toward specific microhabitat refugia alongside concurrent behavioral suppression—to minimize parasite exposure while conserving basal energy. This research seeks to elucidate how specialized herbivores navigate concurrent energetic and parasitological constraints, providing empirical evidence to optimize enclosure design and improve animal welfare in pre-release conservation programs.

## 2. Materials and Methods

### 2.1. Study Area and Focal Subjects

Fieldwork was conducted from early spring to early autumn (March to September 2022) at the Daxiangling Reintroduction Base, located in the transitional ecotone between the Qinghai–Tibet Plateau and the Sichuan Basin within the eastern Hengduan Mountains (29°32′–29°33′ N, 102°50′–102°51′ E; elevation 2410–2560 m). The study area comprises three independent, fenced, semi-wild enclosures (encompassing 0.14, 0.11, and 0.50 km^2^, respectively) [32,33].

The study cohort consisted of three adult female giant pandas (aged 6 years, *n* = 2; aged 9 years, *n* = 1) undergoing wild-adaptation training. To completely eliminate intra-specific interactions and territorial avoidance, the experimental design strictly adhered to a one-individual-per-enclosure protocol. Each of the three pandas was housed completely independently within one of the three separate, fenced, semi-wild enclosures. To control for physiological and pharmacological confounders that might alter behavioral or immunological baselines, regular veterinary assessments—including voluntary blood sampling for hematological and biochemical profiling—were conducted. All subjects were certified clinically healthy, receiving no antibiotics, hormonal therapies, acaricides, or anthelmintic agents for at least six months prior to and throughout the entire sampling period.

### 2.2. High-Resolution Microhabitat Mapping and Meteorological Monitoring

To precisely quantify the spatial heterogeneity of the parasite risk landscape, a comprehensive microhabitat survey was conducted in June 2022. A contiguous grid system was established across all three enclosures [34]. To strictly account for the inherent Circular Error Probable (CEP) of the GPS telemetry (approximately ±15 m), grid cells were set to 30 m × 30 m, yielding a total of 931 sampling quadrats.

Within each quadrat, five 10 m × 10 m sub-plots (four at the vertices and one central) were systematically surveyed. Data from these five sub-plots were averaged to represent the overall microhabitat profile of each quadrat. Surveyed metrics included topographical features (elevation, slope, aspect, distance to the nearest stream) and fine-scale vegetation architecture (canopy closure, leaf litter depth, as well as the abundance and average height of trees, shrubs, and bamboo). Spatial data digitization and rasterization were performed using ArcGIS Pro (version 3.0, Esri Inc., Redlands, CA, USA).

Concurrently, microclimate dynamics were captured using an automated meteorological station situated immediately adjacent to the enclosures. Environmental parameters (ambient temperature, relative humidity, solar radiation, atmospheric pressure, wind speed, wind gust, and precipitation) were recorded at 1-min intervals. These high-frequency data were subsequently aggregated into daily metrics (e.g., daily mean temperature and cumulative precipitation) to align with both the behavioral and parasitological sampling resolutions.

### 2.3. Behavioral Telemetry and Parasite Quantification

Giant panda spatial behavior was monitored continuously using Global Positioning System (GPS) collars. The telemetry units were programmed for isochronous sampling, recording one spatial fix per hour (yielding 24 temporal locations per individual daily). Over the 7-month observation period, the collars successfully recorded a total of 12,598 valid spatial fixes across the three individuals (averaging 4199 fixes per animal). Based on the theoretical maximum of 15,408 expected fixes (24 fixes/day × 214 days × 3 individuals), the overall fix success rate was 81.8%, indicating highly robust data quality despite the dense canopy and rugged topography. To accurately reflect the physical exertion required in the rugged mountainous terrain, daily movement distances were not calculated as simple two-dimensional (2D) Euclidean vectors. Instead, consecutive hourly GPS coordinates were projected onto a high-resolution Digital Elevation Model (DEM) in ArcGIS Pro to calculate the true three-dimensional (3D) topographical trajectory traversed by each individual daily [35].

Tick parasitism was quantified via standardized daily inspections. Leveraging behavioral habituation protocols established during the soft-release training, researchers conducted close-proximity visual and physical examinations of the pandas daily between 09:00 and 09:30. This strictly standardized morning window was chosen to align with the daily habituation routine—minimizing animal stress—and to eliminate circadian biases, thereby ensuring that each count accurately reflected a consistent 24-h accumulation of parasite exposure. The total count of ticks attached to the highly susceptible aural regions was recorded as the daily parasite load. The aural regions were specifically targeted because their thinner skin, high vascularization, and inaccessibility to self-grooming make them primary attachment sites for ectoparasites on large mammals. While conducting daily physical examinations on conscious animals precludes an absolute whole-body tick census, standardizing the counting area to these target sites provides a highly reliable relative index of environmental parasite exposure. Due to the dense pelage of giant pandas and the logistical constraints of conducting physical examinations on conscious animals, detecting microscopic larvae or nymphs is largely unfeasible; thus, our counts specifically represent adult tick burdens. Morphological identification using standard taxonomic keys (assessing the scutum, basis capituli, and coxal spurs) confirmed that all collected adult ticks belonged to *Ixodes ovatus*, consistent with our prior comprehensive molecular inventories (combining Cytochrome c oxidase subunit I [COI] and 16S rRNA sequencing) of this study site, which established *I. ovatus* as the predominant tick species [36].

### 2.4. Spatiotemporal Integration: Implicit Time-Weighted Exposure

A critical methodological challenge was translating discrete spatial metrics into a biologically meaningful measurement of daily microhabitat exposure. Recognizing that tick acquisition is a probabilistic, cumulative process heavily dependent on host residence time, we developed an implicit time-weighted spatial join algorithm using the geopandas (version 0.13.2) and pandas (version 2.0.3) libraries in Python (version 3.10.12) [17].

For each individual on any given day, the 24 hourly GPS fixes were spatially intersected with the 30 m × 30 m microhabitat grid layer. Rather than calculating a simple spatial average of the unique grids traversed, we computed the arithmetic mean of the microhabitat values corresponding to all 24 temporal fixes. Because the telemetry sampling frequency was strictly isochronous (1 fix/hour), this approach mathematically constitutes an intrinsic time-weighted average. Consequently, a high-risk microhabitat (e.g., characterized by thick leaf litter and high canopy closure) occupied by an individual for 18 h exerted a proportionally heavier mathematical weight on its daily exposure profile than a habitat merely transited for one hour. This aggregated “Daily Mean Exposure” metric rigorously quantifies the cumulative exposure of the host across varying gradients of environmental risk.

### 2.5. Statistical Analyses

To evaluate the efficacy of the hierarchical reduced tick load strategies, data manipulation, statistical modeling, and visualization were conducted using Python (version 3.10.12) and GraphPad Prism (version 9.0). A fundamental principle across all inferential models was the explicit control for pseudoreplication arising from repeated longitudinal observations; thus, individual identity was consistently specified as a clustering variable or random intercept [37].

#### 2.5.1. Parasite Pressure and Climatic Drivers

To characterize temporal infestation dynamics, observation records were aggregated into daily tick counts per individual. The seasonal trajectory was visualized using a 7-day rolling mean to smooth short-term daily stochasticity. To disentangle underlying seasonal trends from immediate meteorological fluctuations, daily tick counts were modeled using Generalized Estimating Equations (GEEs) implemented via the statsmodels library (version 0.14.0) [38]. To accommodate the severe overdispersion inherent in parasite count data, we specified a negative binomial distribution with a log link function and an exchangeable working correlation structure, clustered by individual identity to compute robust standard errors. The full model incorporated the sampling month as a categorical predictor (March as baseline), alongside continuous daily mean temperature and precipitation. Daily relative humidity was excluded to prevent matrix singularity, as it remained saturated at 100% throughout the observation period. Effect sizes were exponentiated and reported as Incidence Rate Ratios (IRRs) with robust 95% confidence intervals (CIs).

#### 2.5.2. Quantification of Microhabitat Niche Shift

To assess spatial avoidance, the daily microhabitat niche was characterized using eight core environmental variables. Following Z-score standardization (scikit-learn version 1.2.2) to prevent disproportionate weighting by variables with larger scales (e.g., elevation), Principal Component Analysis (PCA) was employed to extract the primary axes of ecological variation [39]. Structural divergence of the microhabitat niche between the high-risk window (May–July) and the low-risk baseline was visualized using PCA biplots and group-mean standardized radar charts.

Multidimensional niche divergence was statistically tested using a Permutational Multivariate Analysis of Variance (PERMANOVA; Euclidean distance, 999 permutations). To account for individual spatial preferences and repeated measures, permutations (scipy.spatial version 1.10.1) were constrained within blocks defined by individual identity [40]. Subsequently, daily individual scores from the first principal component (PC1)—representing the primary microhabitat gradient—were extracted for univariate longitudinal modeling. A linear mixed model (LMM) quantified the magnitude of this shift, while a polynomial LMM (incorporating linear and quadratic temporal terms) modeled the continuous, non-linear trajectory of spatial avoidance. For all LMMs utilized in this study, the assumption of normality of residuals was rigorously verified via visual inspection of Q-Q plots and histograms prior to interpretation. The theoretical timing of the maximum microhabitat shift was mathematically derived using the vertex equation (−*b*/2*a*) of the quadratic model.

#### 2.5.3. Behavioral Plasticity and Temporal Dynamics

To evaluate behavioral suppression in response to escalating parasite pressure, we analyzed daily 3D topographical movement distances. Recognizing that such behavioral modification represents a sustained state rather than an acute reaction, a 7-day rolling mean was applied to the movement data. The temporal correlation between smoothed tick counts and movement distances was assessed using a non-parametric Spearman’s rank correlation.

The seasonal trajectory of behavioral suppression was modeled using a polynomial LMM. To isolate this behavioral response from immediate meteorological constraints (i.e., heat or precipitation avoidance), a multiple LMM was constructed. The categorical risk period served as the primary fixed effect, with daily mean temperature and precipitation concurrently fitted as continuous covariates to account for climatic variance.

#### 2.5.4. Adaptive Landscape and Parasitological Outcomes

To evaluate the protective efficacy of the combined spatial-behavioral strategy, analyses were subset to the high-risk season (May–July) to ensure uniform background parasite pressure. A 3-day rolling mean of movement distance was used to define continuous behavioral states (sedentary vs. roaming). Consistent with previous analyses, a multivariate GEE model (negative binomial family, robust standard errors) was constructed to predict daily tick counts, simultaneously utilizing PC1 scores, 3-day rolling movement, and their interaction term as primary predictors, while controlling for meteorological covariates (daily mean temperature and precipitation).

To ensure the robustness of the behavioral metric, extensive sensitivity analyses were performed by re-fitting the GEE models across a continuum of rolling movement windows (2, 3, 4, and 5 days). The predictive marginal effects derived from the optimized model were visualized as a 2 × 2 heatmap using seaborn (version 0.12.2) and matplotlib (version 3.7.1) to delineate the predicted parasitological landscape. Finally, empirical distributions of daily tick counts were segmented into four behavioral–spatial strategy quadrants (based on median splits) and visualized using violin and boxplots generated in GraphPad Prism (version 9.0) to corroborate model predictions with actual field outcomes.

## 3. Results

### 3.1. Tick Parasitism Exhibits a Protracted Summer Peak Independent of Immediate Meteorological Fluctuations

Our field observations revealed a pronounced seasonality in the intensity of tick parasitism on the giant pandas undergoing wild-adaptation training. From the early spring baseline in March (mean = 5.47 ± 0.56 SE ticks/day), the daily tick count exhibited a substantial and continuous increase, culminating in a protracted high-risk window spanning from May to July (Figure 1A, Table S1). During this peak period, the average parasitism intensity remained consistently high, recording 12.16 ± 0.92, 11.53 ± 1.01, and 10.43 ± 0.77 ticks/day in May, June, and July, respectively (Table S1). The raw data distribution further highlighted the overdispersion of parasite counts during mid-summer, characterized by episodic high-infestation events, including a maximum of 51 ticks recorded on a single individual in one day in June (Figure 1B). Notably, the daily mean relative humidity within the study area remained saturated at a constant 100% across the entire observation period (Figure 1A, Table S1).

To disentangle underlying seasonal trends from immediate meteorological fluctuations while accounting for individual host susceptibility, we applied a GEE model clustered by individual identity. The model confirmed that the protracted summer peak was statistically significant (Table S2). Compared to the March baseline, the incidence rate of tick parasitism increased by a factor of 2.386 in May (IRR = 2.386, 95% CI: 1.465–3.884, *p* < 0.001), peaked at a 2.428-fold increase in June (IRR = 2.428, 95% CI: 1.679–3.511, *p* < 0.001), and remained elevated at a 2.283-fold increase in July (IRR = 2.283, 95% CI: 1.627–3.200, *p* < 0.001) (Figure 1C). Furthermore, after controlling for this underlying biological seasonality, immediate fluctuations in daily mean temperature (*p* = 0.420) and precipitation (*p* = 0.716) showed no significant effects on tick counts (Figure 1C, Table S2).

### 3.2. Spatiotemporal Divergence of Microhabitat Niche Corresponds with Peak Parasite Pressure

Ordination of the eight standardized microhabitat variables via PCA revealed a structural divergence in habitat utilization between the high-risk (May–July) and low-risk periods (Figure 2A). This multivariate niche shift was statistically corroborated by a Permutational Multivariate Analysis of Variance (PERMANOVA) on the full 8-dimensional microhabitat matrix. The overall microhabitat niche during the high-risk window shifted significantly from the low-risk baseline (Pseudo-F = 2.453). This structural divergence remained statistically significant when permutations were constrained within individual identity to account for repeated measures (*p* = 0.010, Table S4A). The primary ecological gradient extracted by PCA (PC1) explained 34.57% of the total variance. PC1 was strongly driven by positive loadings for canopy closure (0.831), leaf litter depth (0.778), and tree abundance (0.761), contrasting with negative loadings for elevation (−0.485) and bamboo abundance (−0.586, Table S3). Biologically, this PC1 axis clearly represents a structural habitat gradient transitioning from low-elevation, thick-litter woodlands to high-elevation bamboo refugia. Additionally, the second principal component (PC2), which explained 22.89% of the variance, was primarily driven by elevation (0.818) and bamboo abundance (0.741), further underscoring the altitudinal divergence in spatial utilization (Table S3). Visualization of the standardized microhabitat profiles further illustrated this multidimensional shift. Compared to the low-risk baseline, the utilization polygon during the high-risk window expanded towards higher elevations and steeper slopes, while concurrently contracting along the axes of leaf litter depth, canopy closure, and shrub abundance (Figure 2D). Furthermore, the distinct sub-clustering of data points observed within the PCA ordination space (Figure 2A) biologically reflects the highly patchy distribution of these divergent microhabitat types within the montane forest ecosystem.

LMM indicated a trend of individuals shifting along this primary ecological gradient (i.e., towards lower PC1 scores) during the high-risk window (PC1 Estimate = −0.186, *p* = 0.090, Table S4B). To assess the temporal trajectory of this spatial shift, we fitted a polynomial LMM across the observation period. The model revealed a statistically significant quadratic trend (Month^2^: Estimate = 0.043, Z = 2.74, *p* = 0.006), manifesting as a sustained decrease in PC1 scores throughout the summer months (Figure 2B). Derivation of the quadratic vertex (−*b*/2*a*) indicated that the theoretical maximum shift toward these specific microhabitats occurred at month 5.16 (early-to-mid May), temporally coinciding with the onset of peak tick parasitism. Similarly, LMM indicated a highly significant shift along the secondary ecological gradient (i.e., towards higher PC2 scores, representing higher elevations and denser bamboo patches) during the high-risk window (PC2 Estimate = 0.139, *p* < 0.001, Table S4C). To assess the temporal trajectory of this secondary spatial shift, we fitted an additional polynomial LMM. The model revealed a highly significant quadratic trend (Month^2^: Estimate = −0.034, Z = −9.98, *p* < 0.001), manifesting as a sustained increase in PC2 scores that peaked during the summer months (Figure 2C). Derivation of the quadratic vertex (−*b*/2*a*) indicated that the theoretical maximum shift toward these specific microhabitats occurred at month 7.33 (early-to-mid July), temporally coinciding with the peak summer temperatures and maximum environmental parasite pressure. Together, PC1 and PC2 explain 57.46% of the total variance, strongly corroborating the multidimensional nature of this spatial avoidance strategy.

### 3.3. Behavioral Suppression Temporally Coincides with Peak Parasite Exposure

In conjunction with spatial niche shifts, individuals exhibited behavioral plasticity in their daily activity levels during periods of elevated parasite counts. Time-series visualization with 7-day rolling means illustrated an inverse temporal relationship between parasite load and daily movement distance throughout the observation period (Figure 3A). This inverse relationship was statistically supported by a Spearman’s rank correlation analysis, which demonstrated a negative correlation between tick counts and daily movement distance (Spearman r = −0.140, *p* = 0.041, Figure 3B).

Consequently, analysis of daily movement distance revealed a seasonal suppression during the summer months. A polynomial LMM, controlling for individual identity, revealed a statistically significant quadratic trend in movement distance over the observation period (Month^2^: *p* < 0.001, Figure 3C). The mathematical minimum of this behavioral trajectory occurred in early July (month 7.17), temporally coinciding with the sustained peak of tick parasitism.

To disentangle this behavioral response from confounding meteorological factors (e.g., avoiding heat or precipitation), a multiple LMM was constructed. After controlling for daily mean temperature (*p* < 0.001) and precipitation (*p* = 0.505), the high-risk window remained a statistically significant independent predictor, associated with a reduction in daily movement by an average of 103.87 m (Z = −2.96, *p* = 0.003) compared to the low-risk baseline (Table S5).

### 3.4. Microhabitat Selection Determines Parasite Exposure During Peak Infestation Periods

To evaluate the protective efficacy of the observed spatial and behavioral strategies, daily tick counts during the high-risk season (May–July) were modeled using a GEE framework. The model identified microhabitat selection (PC1) as the primary determinant of parasite exposure. Shifting toward specific microhabitats associated with lower PC1 scores significantly decreased daily tick burdens (Estimate = 0.041, Robust SE = 0.010, Z = 3.982, *p* < 0.001; Table S6). Within this seasonal period of generalized behavioral suppression, the independent continuous effect of the 3-day rolling movement distance (Estimate = −0.008, Robust SE = 0.008, Z = −1.008, *p* = 0.314) and its interaction with the PC1 gradient (Estimate = 0.000, Robust SE = 0.002, Z = 0.091, *p* = 0.927) were not statistically significant (Table S6). Sensitivity analyses utilizing 2-day, 4-day, and 5-day rolling movement windows consistently supported the statistically significant main effect of spatial microhabitat selection across all tested temporal scales (all *p* < 0.01; Table S6).

Predictive marginal effects derived from the optimized model were evaluated across four distinct behavioral–spatial strategy quadrants. The heatmap demonstrated that occupying microhabitats with lower PC1 scores minimized predicted parasite loads irrespective of the concurrent behavioral state (yielding 11.2 to 11.3 predicted ticks per day; Figure 4A). In contrast, remaining sedentary within high-risk microhabitats (higher PC1 scores) produced the highest predicted daily parasite load (12.1 ticks per day; Figure 4A). The empirical probability distribution of the raw daily tick counts across these four quadrants aligned with the model predictions, demonstrating that episodic high-infestation events predominantly occurred within high-risk microhabitats regardless of movement intensity (Figure 4B).

## 4. Discussion

Our analytical models reveal that fine-scale microhabitat selection operates as the primary determinant of parasite exposure during the high-risk summer season, significantly outweighing the independent effect of behavioral movement suppression. Specifically, predictive marginal effects demonstrated that utilizing spatial refugia—characterized by higher elevations, dense bamboo coverage, and minimal leaf litter depth—significantly minimized daily tick burdens. Conversely, remaining in habitats situated at lower elevations with thick leaf litter accumulation and sparse understory vegetation resulted in the highest predicted parasite loads, irrespective of the concurrent behavioral state. These empirical outcomes indicate that precise spatial positioning provides a fundamental ecological barrier against seasonal parasitism for giant pandas undergoing semi-wild adaptation. From an applied behavior perspective, this demonstrates that providing access to structurally complex microhabitats is more critical for empowering animals to autonomously manage parasite risk than relying on their localized activity reduction, offering direct implications for pre-release enclosure design [3,4].

The primary protective effect of these specific microhabitats aligns directly with the physiological constraints of questing ectoparasites. Tick species within the genus *Ixodes* are highly susceptible to desiccation and depend heavily on the humid microclimates provided by decaying leaf litter for survival and rehydration [41,42]. By migrating to higher elevations characterized by thinner leaf litter layers and denser bamboo coverage, giant pandas relocate to environments with substantially lower microclimatic humidity, thereby suppressing tick questing efficiency [43,44]. Similar spatial risk-reduction strategies occur in wild ungulates such as European roe deer (*Capreolus capreolus*) and Arctic reindeer (*Rangifer tarandus*), which selectively avoid specific woodland patches or migrate to high-elevation snow patches during peak ectoparasite seasons [14,45]. Our findings suggest that managed individuals retain these natural spatial evasion behaviors when provided with appropriate environmental heterogeneity. Previous ecological assessments concluded that giant pandas migrate to higher elevations during summer primarily for thermoregulation [29]. The robustness of this spatial avoidance strategy is statistically corroborated by our multidimensional microhabitat analysis. While the primary ecological gradient (PC1) captures the structural avoidance of thick-litter woodlands, the secondary gradient (PC2) distinctly reflects a targeted altitudinal shift towards higher-elevation bamboo refugia. Together, these two spatial axes capture 57.46% of the environmental variance, underscoring that the pandas’ response to peak summer parasite pressure is not merely a unidirectional retreat, but a highly complex, multidimensional habitat optimization. Our empirical results provide a complementary parasitological perspective. The simultaneous reduction in ambient temperature and tick questing suitability at higher elevations suggests that thermoregulatory demands and reduced tick load defenses act as mutually reinforcing mechanisms driving the observed microhabitat niche shift. Facilitating this dual-benefit behavioral shift is therefore vital for maintaining both physiological and psychological animal welfare during pre-release training [30,31,46].

Within this high-risk temporal window, the continuous reduction in movement distance did not yield an independent statistical effect on tick counts, reflecting a generalized state of baseline summer hypoactivity across all monitored subjects. While the exceptionally low energy expenditure inherent to their obligate bamboo diet provides a metabolic baseline for this uniform behavioral suppression [28], this profound summer lethargy may also be functionally intertwined with specific reproductive physiological rhythms [47,48]. In adult female giant pandas, the summer months frequently coincide with prolonged elevations in progesterone associated with spontaneous ovulation and pseudopregnancy, a state characterized by significantly reduced dietary intake and stationary nesting behaviors [49]. Although our study did not encompass endocrine monitoring to empirically validate this link, the temporal synchronization between this potential hormone-driven lethargy and the environmental peak of *Ixodes* tick questing presents a critical consideration for health and welfare management in pre-release breeding programs [26,50,51]. By remaining stationary within specific, drier, high-elevation microhabitats during this critical reproductive phase, female giant pandas might incidentally acquire substantial parasitological protective benefits without expending additional energy. This physiological and ecological synchronization ensures that energetic conservation efforts do not result in severe parasitic hyper-infestation. For conservation managers, this implies that enclosures lacking these specific structural refugia could expose lethargic females to severe welfare risks, similar to challenges observed in denning black bears (*Ursus americanus*) avoiding ectoparasites during periods of metabolic depression [52].

The strong reliance on high-elevation refugia for simultaneous thermoregulatory and parasitological defense raises critical conservation concerns under global climate change [53]. Anthropogenic warming is shifting the geographic distribution of *Ixodes* ticks toward higher latitudes and altitudes worldwide [54]. If sustained climate change facilitates the vertical expansion of tick populations into current summer refugia, the spatial barrier identified in this study will be severely compromised. The altitudinal compression of these refugia threatens to expose giant pandas to unprecedented parasite loads during a season of pronounced physiological vulnerability [55]. Incorporating expansive, heterogeneous landscapes and intact altitudinal gradients into the design of future pre-release training bases is essential to ensure managed populations retain accessible microhabitat options to mitigate escalating parasitological threats, thereby safeguarding animal welfare in a rapidly changing climate [19,56].

A fundamental challenge in evaluating wildlife spatial behavior is disentangling the primary drivers of localized movement, particularly given the natural multicollinearity between parasitological risk, thermoregulatory demands, and foraging phenology. It is highly probable that the macro-scale altitudinal shifts observed during the summer are simultaneously driven by the pursuit of emerging bamboo shoots at higher elevations and the necessity to escape heat stress in the valleys. However, our inferential models controlled for daily macro-scale meteorological variables (temperature and precipitation). More importantly, the reduced tick load benefits were statistically bound to the fine-scale microhabitat architecture (e.g., leaf litter depth and specific canopy closure) rather than mere altitudinal displacement. Therefore, while nutritional and thermoregulatory requirements may dictate the macro-geographical boundaries of their summer range, precise microhabitat selection within these zones functions as an indispensable, synergistic mechanism to mitigate parasite exposure. Future applied frameworks should integrate high-resolution bamboo phenology mapping and animal-borne microclimate loggers (e.g., body temperature and local humidity sensors) to quantitatively partition the relative ecological drivers of foraging, thermoregulation, and parasite avoidance within managed landscapes. Additionally, several methodological limitations necessitate cautious interpretation and provide clear directions for future research. The sample size was restricted to three individuals, and the cohort consisted exclusively of adult females. Because the observed summer lethargy might be confounded by female-specific physiological cycles (such as unmeasured pseudopregnancy events), extrapolating this baseline behavioral suppression to adult males or juveniles requires further validation [57]. The absence of concurrent endocrine monitoring (e.g., non-invasive fecal steroid hormone analysis) precludes a definitive quantitative linkage between progesterone fluctuations and the observed spatial and behavioral shifts [58]. Crucially, while the subjects resided in fenced semi-wild adaptation enclosures—which inherently restricts broad-scale geographic dispersion—this context perfectly represents the actual spatial constraints faced by animals undergoing pre-release training. In completely wild settings, giant pandas can undertake extensive macro-scale altitudinal migrations across unrestricted home ranges to evade severe environmental stressors. In contrast, the spatial constraints imposed on semi-wild individuals preclude such broad-scale geographic evasion, thereby forcing a heavier reliance on fine-scale behavioral plasticity and precise microhabitat selection to combat seasonal tick infestations. Rather than merely limiting the observable spatial avoidance capabilities of the species, this setting provides highly applicable baseline data for optimizing the size and structural composition of conservation enclosures [59]. Finally, this study evaluated the avoidance of a single dominant tick genus. However, giant pandas may encounter a diverse assemblage of sympatric ectoparasites during the summer, including haemadipsoid leeches and biting dipterans [60,61]. From a behavioral ecology perspective, ticks largely employ a strategy of unobtrusiveness (painless attachment), making the formation of a direct ‘Pavlovian’ associative avoidance strictly towards ticks somewhat challenging [62]. It is highly plausible that the immediate painful stimuli and annoyance inflicted by swarming, conspicuous pests (e.g., mosquitoes and horseflies) serve as the primary behavioral triggers that drive pandas to seek cooler, windier, and higher-elevation bamboo refugia [63]. Because the seasonal dynamics and environmental drivers of these conspicuous tormentors largely overlap with those of *Ixodes ovatus*, the observed tick avoidance may functionally represent a “co-avoidance” strategy—a highly beneficial secondary byproduct of escaping a broader ectoparasite community. Future research should employ multi-parasite community analyses to further disentangle these complex host–parasite–environment interactions [22,26,27,64,65].

## 5. Conclusions

This study elucidates the complex behavioral and spatial mechanisms giant pandas employ to navigate peak seasonal parasite exposure within strict energetic constraints during semi-wild adaptation. We demonstrate that reduced tick load defense in this specialized herbivore is hierarchically structured. While behavioral suppression serves as a seasonal baseline response to escalating summer tick parasitism, precise microhabitat selection functions as the primary driver of parasite avoidance. By actively utilizing high-elevation refugia characterized by minimal leaf litter and dense bamboo coverage, individuals construct a critical ecological barrier against environmentally mediated parasitism, effectively minimizing the risk of hyper-infestation. From an applied management perspective, these findings demonstrate that providing fine-scale spatial heterogeneity empowers managed animals to autonomously mitigate health risks, compensating for their inability to undertake energetically costly broad-scale displacement within fenced enclosures. Ultimately, the strong reliance on these specific, high-elevation microhabitats highlights a latent vulnerability to global climate change, presenting a direct challenge for future pre-release programs. As warming temperatures facilitate the vertical expansion of tick distributions, incorporating intact, heterogeneous altitudinal gradients into enclosure designs becomes imperative. Safeguarding the structural complexity of these training environments is no longer solely about securing continuous nutritional resources, but also ensuring that giant pandas retain the spatial resilience required to mitigate escalating parasitological threats, thereby maximizing their welfare and ultimate reintroduction success.

## Figures and Tables

**Figure 1:**
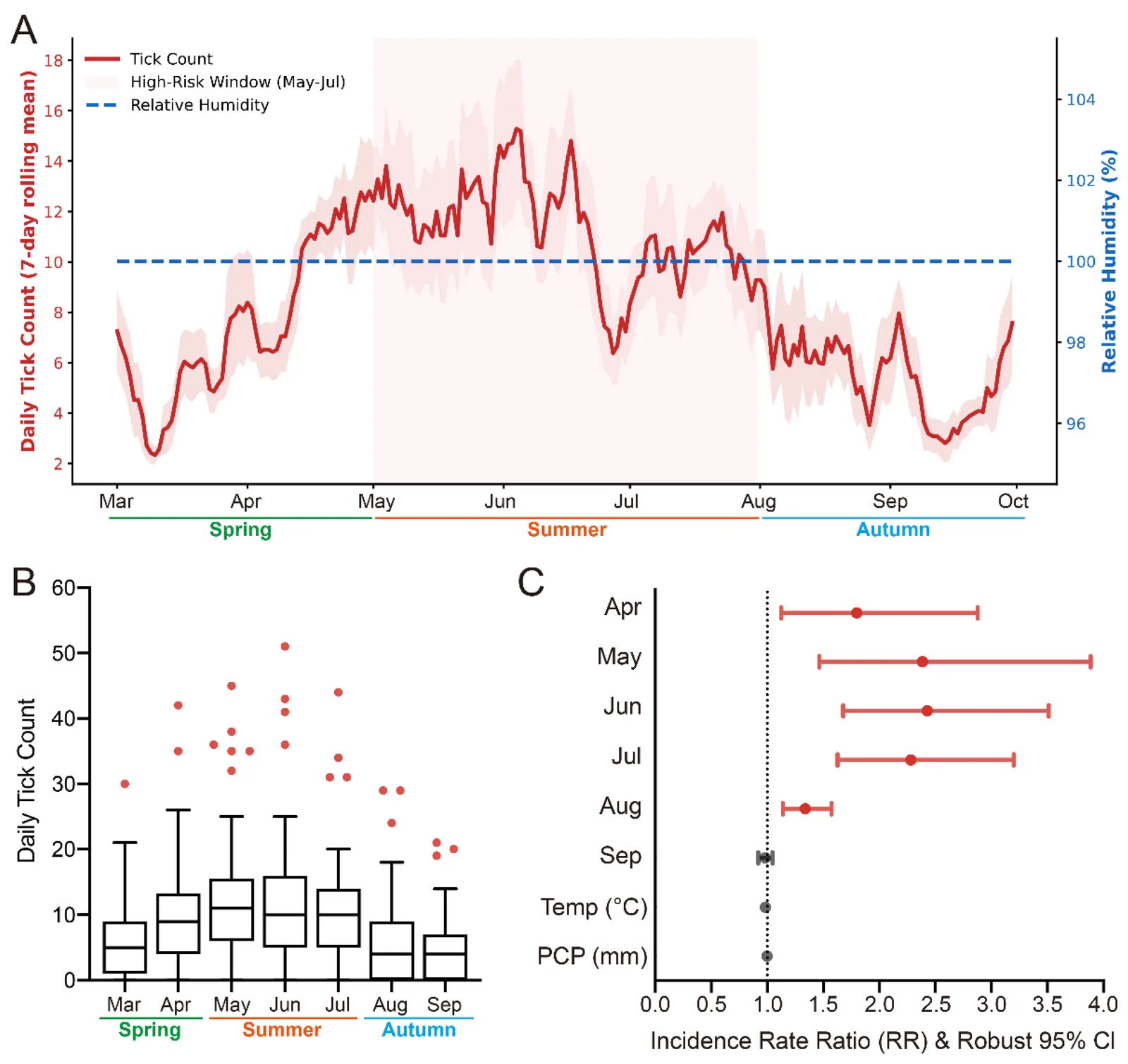
Spatiotemporal dynamics and statistical drivers of tick (*Ixodes ovatus*) parasitism on giant pandas. (**A**) Temporal dynamics of tick parasitism. The solid red line represents the 7-day rolling mean of daily tick counts, flanked by shaded error bands indicating standard deviation. The dashed blue line represents the daily mean relative humidity. The shaded pink region delineates the protracted high-risk window from May to July. (**B**) Seasonal distribution of daily raw tick counts across spring, summer, and autumn. Boxplots depict the median (thick red horizontal line) and interquartile range (IQR). Whiskers are constructed using the Tukey method, extending to the furthest data points within 1.5× IQR. Upper outliers beyond the whiskers are explicitly highlighted as red dots to illustrate episodic high-infestation events. (**C**) Forest plot illustrating the effect sizes of seasonal and climatic drivers on daily tick counts. Data are presented as Incidence Rate Ratios (IRRs) with robust 95% confidence intervals derived from a negative binomial GEE clustered by individual identity. The black dashed line indicates the null effect threshold (IRR = 1.0). Relative humidity was excluded from the model due to its constant saturation at 100%.

**Figure 2:**
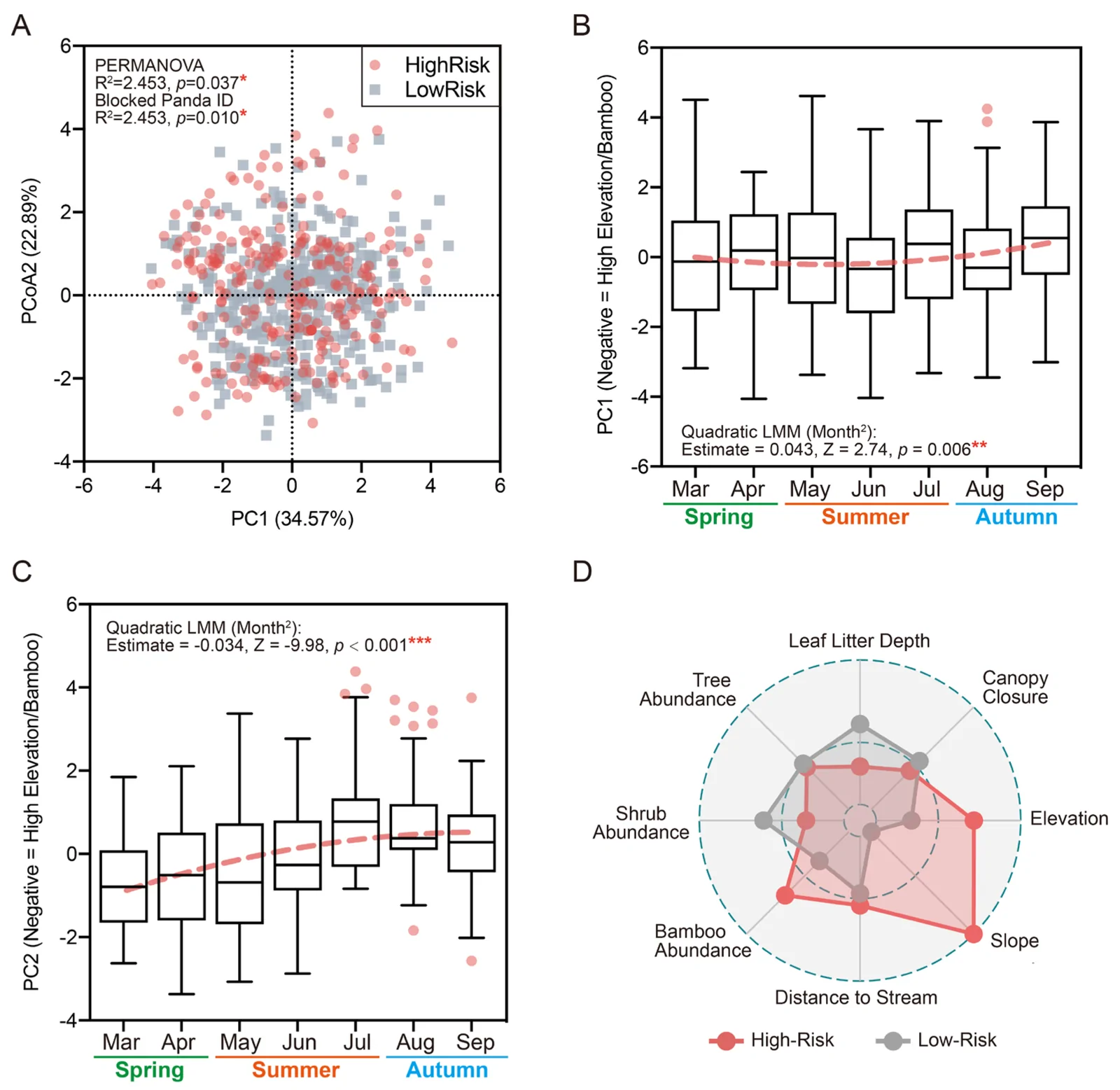
Spatiotemporal shift in microhabitat utilization by giant pandas in response to varying parasite pressure. (**A**) PCA biplot illustrating the divergence of microhabitat niches. Red and grey dots represent daily microhabitat exposures during the high−risk (May−July) and low−risk periods, respectively. Temporal trajectory of the primary (**B**, PC1) and secondary (**C**, PC2) microhabitat gradients across the observation period (labeled by month and season). Boxplots display the median (horizontal line) and IQR. Whiskers are constructed using the Tukey method, extending to the furthest data points within 1.5 × IQR, with upper outliers marked as red dots. The thick red dashed line represents the fitted curve from a polynomial LMM, revealing statistically significant quadratic trend for both PC1 (Month^2^ *p* = 0.006, vertex in early May) and PC2 (Month^2^ *p* < 0.001, vertex in mid−July). (**D**) Radar chart illustrating the standardized microhabitat profiles. The red and grey polygons represent the mean standardized values of each habitat factor during the high-risk and low-risk periods, respectively. Concentric circles, from the inner to the outer bounds, represent standardized scale values of −0.125, 0, and 0.165. Asterisks denote statistical significance (* *p* < 0.05, ** *p* < 0.01, *** *p* < 0.001).

**Figure 3:**
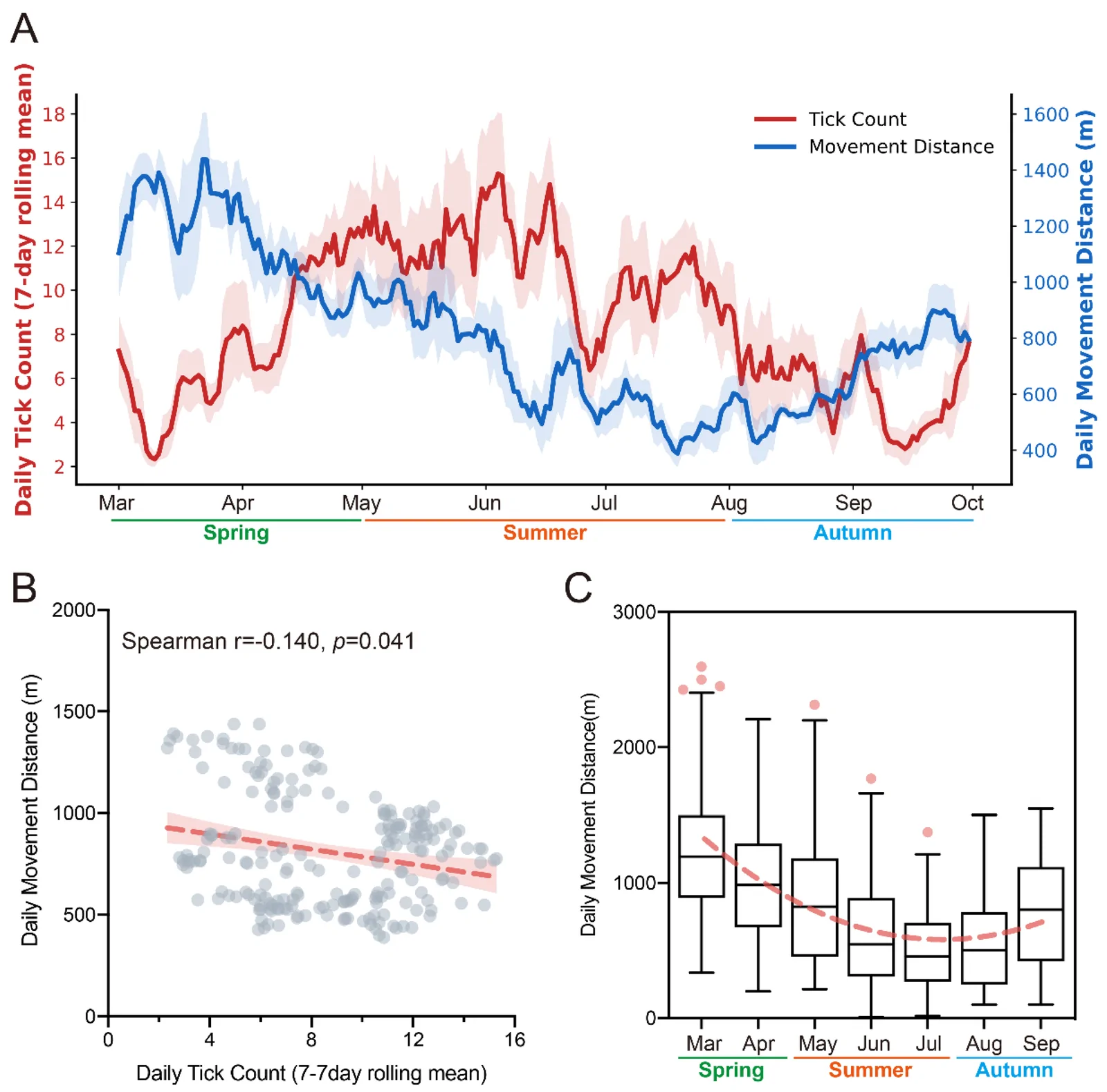
Temporal dynamics of daily movement distance in relation to tick parasitism. (**A**) Inverse dynamic coupling between parasite pressure and movement. The dual Y−axis time series displays the 7−day rolling mean of daily tick counts (red line, left axis) and daily movement distance (blue line, right axis). Shaded bands flanking the curves represent ± 0.5 standard deviation. (**B**) Scatter plot illustrating the negative correlation between daily tick counts and movement distance. Each data point represents the 7−day smoothed daily mean. The thick red dashed line indicates the simple linear regression fit, flanked by the 95% confidence interval shaded band, with significance determined by Spearman’s rank correlation (r = −0.140, *p* = 0.041). (**C**) Temporal trajectory of daily movement distance across the observation period (labeled by month and season). Boxplots display the median and IQR, with Tukey whiskers extending to the furthest data points within 1.5 × IQR, and red dots indicating upper outliers. The thick red dashed line denotes the fitted polynomial curve from a LMM, revealing a statistically significant quadratic seasonal decrease (Month^2^ *p* < 0.001) centered in July.

**Figure 4:**
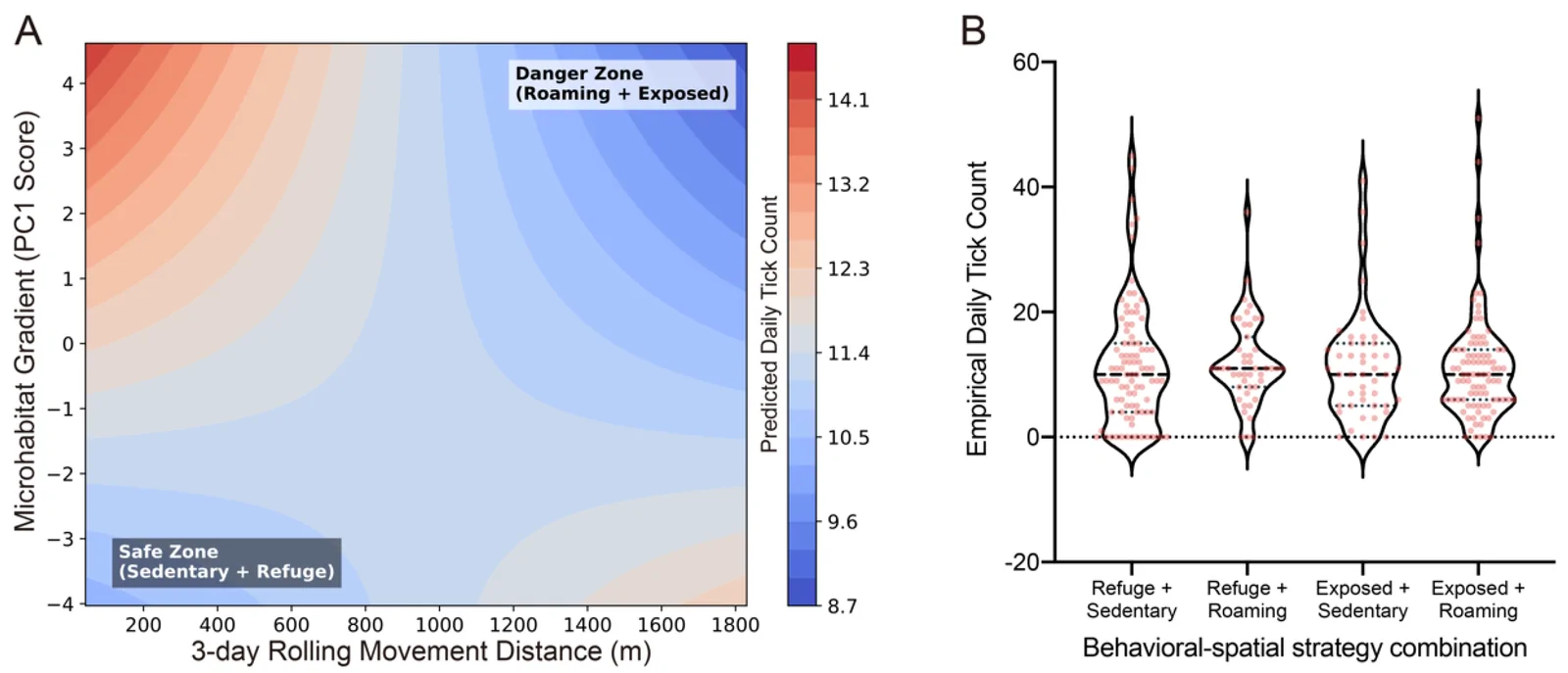
The predicted parasitological landscape and empirical outcomes of spatial−behavioral strategies during the high−risk season (May−July). (**A**) A 2 × 2 heatmap displaying the marginal predicted daily tick counts derived from the GEE model. The quadrants represent combinatorial strategies of microhabitat selection (Low−risk vs. High−risk, determined by median PC1 scores) and behavioral state (Sedentary vs. Roaming, determined by median 3−day rolling movement distances). The model predictions account for meteorological covariates and clustering by individual identity. (**B**) Violin and box plots illustrating the empirical distribution of daily tick counts across the corresponding four strategy quadrants. The violin plots display the probability density of the raw field data, while the internal box plots denote the median (thick horizontal line), IQR (box), and whiskers extending to the furthest data points within 1.5 × IQR.

## Data Availability

The original contributions presented in this study are included in the article/Supplementary Material. Further inquiries can be directed to the corresponding author(s).

## Supplementary Materials

The following supporting information can be downloaded at: https://www.mdpi.com/article/10.3390/ani16193050/s1. Table S1: Descriptive statistics of daily tick (*Ixodes ovatus*) parasitism counts on wild giant pandas and corresponding core climatic variables across different months; Table S2: Results of the Negative Binomial Generalized Estimating Equations (GEE) evaluating the effects of seasonality (Month) and climatic factors on daily tick parasitism counts; Table S3: Principal Component Analysis (PCA) loadings of the 8 microhabitat variables and the proportion of variance explained by the first three principal components (PC1–PC3); Table S4: Statistical tests for microhabitat niche shifts between high-risk and low-risk periods; Table S5: Linear Mixed Model (LMM) disentangling the effects of parasite risk periods and immediate meteorological factors on giant panda daily movement distance (m); Table S6: Sensitivity analysis of the Generalized Estimating Equations (GEE) model across multiple behavioral time windows (2-day, 3-day, 4-day, and 5-day rolling means).
