# Supplementary material for: Microhabitat Selection Drives Tick Avoidance in Giant Pandas During Semi-Wild Adaptation Training: Implications for Conservation Management
Source: Animals (Basel). 2026 Sep 28;16(19):3050. doi: 10.3390/ani16193050 (PMC13663322; doi:10.3390/ani16193050)
Supplement: Supplementary file 1 [file animals-16-03050-s001.zip › animals-4565404-supplementary.pdf]

Microhabitat selection drives tick avoidance in giant pandas during semi-wild adaptation training: Implications for conservation management

Rui Ma[1], Xiang Yu[1], Chong Huang[1], Fei Xue[1], Yanshan Zhou[1], Jiabin Liu[1], Rong Hou[1], Wenlei Bi[1], Jiang Gu[1], Long Zhang[1], Feifei Feng[1], Guanwei Lan[1], Zusheng Li[1], Wei Wu[1], Hong Yang[2], Dunwu Qi[1]*

[1] The Conservation of Endangered Wildlife Key Laboratory of Sichuan Province, Chengdu Research Base of Giant Panda Breeding, Chengdu 510081, China.

[2] Daxiangling Nature Reserve, Yaan, Sichuan 625000, China.

*** Corresponding authors**

Dunwu Qi

Email address: qidunwu@163.com

Table. S1 Descriptive statistics of daily tick (*Ixodes ovatus*) parasitism counts on wild giant pandas and corresponding core climatic variables across different months. Values are presented as Mean, Standard Error (SE), Minimum, and Maximum. High-risk window is identified from May to July.

Month	N (Days)	Mean Ticks	SE	Min Ticks	Max Ticks	Mean RH (%)	Mean Temp (°C)
3	93	5.47	0.56	0	30	100	5.1
4	90	9.52	0.8	0	42	100	6.4
5	93	12.16	0.92	0	45	100	8.9
6	90	11.53	1.01	0	51	100	13.3
7	93	10.43	0.77	0	44	100	16
8	93	6.03	0.67	0	29	100	16.5
9	90	4.87	0.52	0	21	100	10.9

Table. S2 Results of the Negative Binomial Generalized Estimating Equations (GEE) evaluating the effects of seasonality (Month) and climatic factors on daily tick parasitism counts, clustering by Panda ID to obtain robust standard errors.

Parameter	Estimate (Log-Odds)	Robust Std. Error	z value	P-value	95% CI Lower	95% CI Upper	IRR
Intercept	1.781	0.128	13.91	<0.001 ***	1.53	2.032	5.938
Month 4	0.588	0.239	2.45	0.014 *	0.118	1.057	1.8
Month 5	0.87	0.249	3.5	<0.001 ***	0.382	1.357	2.386
Month 6	0.887	0.188	4.71	<0.001 ***	0.518	1.256	2.428
Month 7	0.825	0.173	4.78	<0.001 ***	0.487	1.163	2.283
Month 8	0.292	0.082	3.54	<0.001 ***	0.131	0.454	1.339
Month 9	-0.019	0.033	-0.58	0.561	-0.083	0.045	0.981
Mean Temperature (°C)	-0.016	0.02	-0.81	0.42	-0.055	0.023	0.984
Precipitation (mm)	-0.001	0.004	-0.36	0.716	-0.008	0.006	0.999

Note: Relative humidity was excluded due to its constant saturation (100%). Month 3 is set as the reference category. IRR stands for Incidence Rate Ratio. Significance codes: *** < 0.001, ** < 0.01, * < 0.05.

Table. S3 Principal Component Analysis (PCA) loadings of the 8 microhabitat variables and the proportion of variance explained by the first three principal components (PC1–PC3). Variables with absolute loading values > 0.50 on PC1 or PC2 indicate primary drivers of habitat selection.

Microhabitat Variables	PC1	PC2	PC3
Leaf Litter Depth	0.778	0.52	-0.253
Canopy Closure	0.831	0.27	0.093
Elevation	-0.485	0.818	-0.078
Slope	-0.181	0.361	0.69
Distance to Stream	0.041	-0.126	0.536
Bamboo Abundance	-0.586	0.741	-0.22
Shrub Abundance	0.532	0.333	0.576
Tree Abundance	0.761	0.125	-0.33
Explained Variance (%)	34.57%	22.89%	16.61%

Table S4. Statistical tests for microhabitat niche shifts between high-risk and low-risk periods.

(A) PERMANOVA (ADONIS)on the 8-dimensional standardized microhabitat matrix.

Test Type	Distance Metric	Pseudo-F	P-value
Unrestricted Permutations (N=999)	Euclidean	2.453	0.037*
Restricted Permutations (Block = Panda ID, N=999)	Euclidean	2.453	0.010*

(B) Linear Mixed Model (LMM) testing the shift along the primary ecological gradient (PC1).

Parameter	Estimate	Std. Error	z value	P-value
Intercept (Low-Risk Baseline)	0.08	0.661	0.121	0.903
High-Risk Period Effect	-0.186	0.11	-1.697	0.090°
Random Effect (Panda ID Variance)	1.296			

(C) Linear Mixed Model (LMM) testing the shift along the secondary ecological gradient (PC2).

Parameter	Estimate	Std. Error	z value	P-value
Intercept (Low-Risk Baseline)	-0.123	0.199	-0.616	0.538
High-Risk Period Effect	0.139	0.024	5.766	<0.001 ***
Random Effect (Panda ID Variance)	0.119			

Note: Statistical tests evaluating the divergence of microhabitat niches between high-risk and low-risk periods. (A) Permutational Multivariate Analysis of Variance (PERMANOVA/ADONIS) on the full 8-dimensional standardized microhabitat matrix. Results are shown for both unrestricted permutations and restricted permutations (blocking by Panda ID to control for pseudoreplication). (B) Linear Mixed Model (LMM) testing the magnitude of the shift along the primary ecological gradient (Principal Component 1), with

Panda ID fitted as a random intercept. (C) Linear Mixed Model (LMM) testing the magnitude of the shift along the secondary ecological gradient (Principal Component 2). Significance codes: *** < 0.001, * < 0.05, ° < 0.10.

Table. S5 Linear Mixed Model (LMM) disentangling the effects of parasite risk periods and immediate meteorological factors on giant panda daily movement distance (m).

Parameter	Estimate	Std. Error	z value	P-value
Intercept (Low-Risk Baseline)	1314.99	125.56	10.47	<0.001 ***
High-Risk Period Effect	-103.87	35.05	-2.96	0.003 **
Mean Temperature (° C)	-41.03	3.49	-11.76	<0.001 ***
Precipitation (mm)	-0.86	1.28	-0.67	0.505
Random Effect (Panda ID Variance)	42187.05			-

Note: Panda ID was fitted as a random intercept to account for individual baseline differences in activity levels. The Low-Risk period serves as the reference category. Significance codes: *** < 0.001, ** < 0.01, * < 0.05.

Table. S6 Sensitivity analysis of the Generalized Estimating Equations (GEE) model across multiple behavioral time windows (2-day, 3-day, 4-day, and 5-day rolling means).

Parameter	Estimate	Robust Std. Error	z value	P-value
Time Window: 2-day Rolling				
Intercept	2.862	0.396	7.231	<0.001 ***
PC1 (Microhabitat Score)	0.034	0.008	3.963	<0.001 ***
2-day Movement (per 100m)	-0.011	0.006	-1.735	0.083
Mean Temp (° C)	-0.002	0.003	-0.736	0.462
Precipitation (mm)	-0.028	0.033	-0.85	0.395
PC1 × 2-day Movement	0	0.002	0.123	0.902
Time Window: 3-day Rolling				
Intercept	2.839	0.38	7.474	<0.001 ***
PC1 (Microhabitat Score)	0.041	0.01	3.982	<0.001 ***
3-day Movement (per 100m)	-0.008	0.008	-1.008	0.314
Mean Temp (° C)	-0.004	0.004	-1.069	0.285
Precipitation (mm)	-0.027	0.032	-0.851	0.395
PC1 × 3-day Movement	0	0.002	0.091	0.927
Time Window: 4-day Rolling				
Intercept	2.842	0.378	7.516	<0.001 ***
PC1 (Microhabitat Score)	0.039	0.011	3.613	<0.001 ***
4-day Movement (per 100m)	-0.008	0.009	-0.875	0.381
Mean Temp (° C)	-0.004	0.005	-0.862	0.389

Precipitation (mm)	-0.027	0.032	-0.856	0.392
PC1 × 4-day Movement	0	0.002	0.094	0.925
Time Window: 5-day Rolling				
Intercept	2.826	0.355	7.97	<0.001 ***
PC1 (Microhabitat Score)	0.042	0.014	3.071	0.002 **
5-day Movement (per 100m)	-0.006	0.011	-0.581	0.562
Mean Temp (° C)	-0.005	0.006	-0.884	0.377
Precipitation (mm)	-0.027	0.031	-0.869	0.385
PC1 × 5-day Movement	0	0.002	0.046	0.963

Note: Models evaluate the interactive effects of microhabitat selection (PC1) and behavioral suppression on daily tick parasitism during the high-risk season (May-July). The consistent high significance of PC1 across all temporal scales confirms the robustness of spatial refuge utilization as the primary driver of parasite evasion. Significance codes: *** < 0.001, ** < 0.01, * < 0.05.
